# Posterior AD-Type Pathology: Cognitive Subtypes Emerging from a Cluster Analysis

**DOI:** 10.1155/2014/259358

**Published:** 2014-06-05

**Authors:** Antonella Cappa, Nicoletta Ciccarelli, Eleonora Baldonero, Marialuisa Martelli, Maria Caterina Silveri

**Affiliations:** ^1^Department of Geriatrics, Centre for the Medicine of the Ageing, Neurosciences and Orthopedics, Catholic University, Rome, Italy; ^2^Dementia Center, Italian Hospital Group, Via Tiburtina 188, Guidonia, 00012 Rome, Italy; ^3^Institute of Clinical Infectious Diseases, Catholic University, 00168 Rome, Italy; ^4^Department of Psychology, Sapienza University of Rome, 00184 Rome, Italy; ^5^Neuropsychology Unit, IRCCS Fondazione Santa Lucia, 00136 Rome, Italy

## Abstract

*Background*. “Posterior shift” of the neuropathological changes of Alzheimer's disease (AD) produces a syndrome (posterior cortical atrophy) (PCA) dominated by high-level visual deficits. *Objective*. To explore in patients with AD-type pathology whether a data-driven analysis (cluster analysis) based on neuropsychological findings resulted in the emergence of different subgroups of patients; in particular to find out whether it was possible to identify patients with visuospatial deficits consistent with the hypothesis that PCA is a “dorsal stream” syndrome or, rather, whether there were subgroups of patients with different types of impairment within the high-level visual domain. *Methods*. 23 PCA and 16 DAT patients were studied. By a principal component analysis performed on a wide range of neuropsychological tasks, 15 variables were obtained that loaded onto five main factors (memory, language, perceptual, visuospatial, and calculation) which entered a hierarchical cluster analysis. *Results*. Four clusters of cognitive impairment emerged: visuospatial/perceptual, memory, perceptual/calculation, and language. Only in the first cluster a visuospatial deficit clearly emerged. *Conclusions*. AD pathology produces not only variants dominated by memory (DAT) and, to a lesser extent, visuospatial deficit (PCA), but also other distinct syndromic subtypes with disorders in visual perception and language which reflect a different vulnerability of specific functional networks.

## 1. Introduction

The “posterior shift” of the neuropathological changes of Alzheimer's disease (AD) [1] produces a syndrome dominated by high-level visual deficits [2] that on clinical ground is defined as posterior cortical atrophy (PCA). Previous studies [3, 4] comparing the clinical and neuropsychological profile of patients with PCA to a cohort of patients with typical (amnesic) AD concluded that PCA is predominantly a “dorsal stream” syndrome [5, 6]. The major involvement of dorsal visual progressing stream in PCA has been also suggested by Tsai et al. [7] in a retrospective study using a hierarchical cluster analysis to separate 30 PCA patients into dorsal and ventral stream subgroups. Other studies, however, have proposed a distinction in occipitotemporal and biparietal variants of PCA [8], although in many patients features of both variants can coexist [9]. In addition, Galton et al. [10] proposed a third kind of presentation of PCA characterized by an impairment of basic perceptual abilities reflecting the involvement of primary visual cortex.

Neuroimaging techniques have shown a predominantly right posterior hemisphere involvement, compared to a more left-sided pattern observed in typical expression of Alzheimer's dementia [11, 12]. Comparing the distribution of atrophy in various “posterior” clinical syndromes [13], a large overlapping of the atrophy in a temporoparietal network has been demonstrated, in support of the hypothesis that all these syndromes are generated by the same pathology. Recently, Lehmann et al. [14] found specific patterns of hypometabolism (FDG-PET) in clinical variants of early onset AD, including PCA, confirming that AD syndromic expressions are produced by degeneration of specific functional networks.

In this study, 39 patients with probable AD-type pathology, 23 patients who responded to the clinical criteria for PCA [3, 15], and 16 patients who responded to the clinical criteria for dementia of Alzheimer's type (DAT) [16, 17] underwent an extensive neuropsychological examination. The aim of the study was to explore whether a data-driven analysis (cluster analysis) exclusively based on neuropsychological findings resulted in the emergence of different subgroups of patients; in particular to find out whether, apart from patients with typical memory disorders, it was possible to identify patients with visuospatial deficits consistent with the hypothesis that PCA is a “dorsal stream” syndrome [3, 4, 7] or, rather, whether there were subgroups of patients with different types of impairment within the high-level visual domain.

## 2. Materials and Methods

### 2.1. Patients

Twenty-three PCA patients according to the proposed criteria [3, 15] were consecutively enrolled from the ones referred to the Neuropsychological Service of the Centre for the Medicine of the Aging of the Catholic University of Rome, from 2005 to 2010. PCA patients underwent the first clinical assessment complaining of visual dysfunction (11/23; 47,8%), spatial disorientation (6/23; 26,1%), memory deficits (17/23; 73,9%), and depression (5/23; 21,7%). Visuospatial impairment was not attributable to primary visual perception deficits. Twenty (86,9%) PCA patients exhibited some or all elements of Gerstmann's syndrome (agraphia, acalculia, and digital agnosia), 18 (78,3%) PCA patients showed some or all elements of Balint's syndrome (simultanagnosia and oculomotor apraxia), 7 (30,4%) PCA patients presented neglect, and 1 (4,3%) PCA patient showed aphasia. Eight (34,8%) PCA patients presented a presenile onset of the disease. Sixteen DAT patients selected on the basis of standard criteria [16, 17], consecutively enrolled in 2009, also entered the study. At the first clinical assessment DAT patients complained of memory deficits (15/16; 93,7%), depression (2/16; 12,5%), and language deficits (2/16; 12,5%). Ten DAT patients presented some or all elements of Gerstmann's syndrome, 5 patients showed simultanagnosia, and 3 patients exhibited aphasia. Neglect was not observed in DAT patients. Three (18,7%) DAT patients presented a presenile onset of the disease.

All patients had received, within three months from the clinical diagnosis, structural (MRI) and functional (single photon emission computed tomography (SPECT)) examination. No patient presented lesions attributable to pathologies other than atrophic damage. No patient presented history of psychiatric disorder or drug or alcohol abuse.

Table 1 provides demographic and clinical details. DAT and PCA were matched for age (*P* > .5), education (*P* > .5), disease duration (*P* > .5), and clinical and cognitive severity of dementia (clinical dementia rating (CDR): *P* > .5; MMSE: *P* > .5). Patients (or their relatives) gave their informed consent and the study was approved by the Institutional Ethics Committee of the Catholic University of Rome.

### 2.2. Clinical and Neuropsychological Assessment

Subjects underwent an extensive neuropsychological examination (for tasks and references see supplementary materials available online at http://dx.doi.org/10.1155/2014/259358) administrated by a psychologist blinded to clinical information. Twenty-six tasks exploring memory, perceptual and visuospatial domains, executive abilities, language, and calculation (see Table 2) were selected and used as variables for further analyses. Exclusion criteria for the other tasks were tasks not performed by all subjects, similar or identical tasks belonging to different batteries, and insensitive measures tasks at ceiling or floor for most patients. The SPECT performed during routine examination were converted to analyze format using MRIcro software (http://www.mricro.com) and overlaid on a single-subject template provided in MRIcron [18].

### 2.3. Statistical Analysis

In each task the patient's raw scores have been converted into proportion of correct responses and then logit- transformed [19]. This transformation can be applied to perform parametric analysis on scores that are bounded and restricted to a finite interval (0-1). The transformation has been applied according to the following formula: logit(*p*) = Ln(*p* + *a*) − Ln(1 − (*p* − *a*)), where *a* = 0.01; this brings values numerically closer to probits and avoids infinity outcomes in the case of probabilities of zeros and ones. Indeed, in the case of our distribution the 0 and 1 outcomes across tasks have nonzero probability of occurrence. Such probabilities must in our case be considered as structural in that having a finer testing scale could in principle capture variations and an increase in the number of observations could result in correct estimations of guessing rate. With the aim to isolate selective impairments assessed through the tasks that would enable a distinction in the phenotypical expressions of AD independently from severity, we borrowed from the psychometric literature a statistical procedure, the data deflation, that has been applied to solve the problem of an idiosyncratic use of the scale, namely,* response style* [20]. Thus, for each patient the logit of performance has been converted to* z*-scores with mean and standard deviation calculated separately on each patient's performance across all tasks. After this transformation for each patient the performance in a task is reported relative to the mean performance, set to zero, of the patient (i.e., impaired or spared performance is independent of the overall level of accuracy). This procedure is well suited in our case since it requires having multiple heterogeneous behavioural measures. Note that in this case and in the subsequent analysis, differences across patients are netted from the overall general performance deficit. Thus patients are expected to differ only on the dominant cognitive components of their deficit.

A correlation matrix between the 26 tasks above mentioned (Table 2) was computed on the transformed scores. Tasks that correlated more than 0.75 (because of exploring the same domains) were excluded from further analysis, leaving 21 tasks; in other words, the tasks were selected to maximize differences across the cognitive components and minimize overlap among different forms of equivalent tests.

DAT and PCA deflated scores entered into a factorial analysis with varimax rotation to evaluate the correlation across the cognitive components measured by the different tasks. A hierarchical agglomerative cluster analysis was carried out on the derived factor scores. Cluster analysis was based on statistical recommendations [21]. Cluster analysis is a classification technique for forming homogeneous groups within complex data sets and the aim of the present study was to assess how many groups can be distinguished just based on the performance obtained in the screening batteries. It must be noted that this analysis is exploratory in nature, and the result of the clustering depends on the similarity and agglomeration method chosen. We performed a hierarchical cluster analysis with squared Euclidean distance as proximity measure and Ward's minimum variance agglomerative method. In the squared Euclidean distance metric the classification is based solely on the pattern of patient's scores on the variable of interest, without taking into account the elevation scores. The analysis of variance was used to compare variables across clusters. Post hoc comparisons were carried out using Fisher LSD method.

## 3. Results

### 3.1. Variables Selection for Cognitive Abilities Assessment

In a preliminary analysis, using an eigenvalue greater than 1, nine factors were selected. Variables that did not show saturations greater than .45 with any factors were eliminated from further analysis. This left 15 variables (Table 3) that loaded onto five main factors, which collectively accounted for 62% of the total variance: memory (accounting for 20% of the variance, eigenvalue 3.7), language (14%, 2.5), perception (11%, 2), visuospatial analysis (9.7%, 1.7), and calculation (8.5%, 1.5) (Table 3).

### 3.2. Cluster Characteristics

Factor scores were entered into a hierarchical cluster analysis to evaluate the presence of different patterns of deficit in the patients' cognitive components estimated by the five factors extracted.

**Figure 1:**
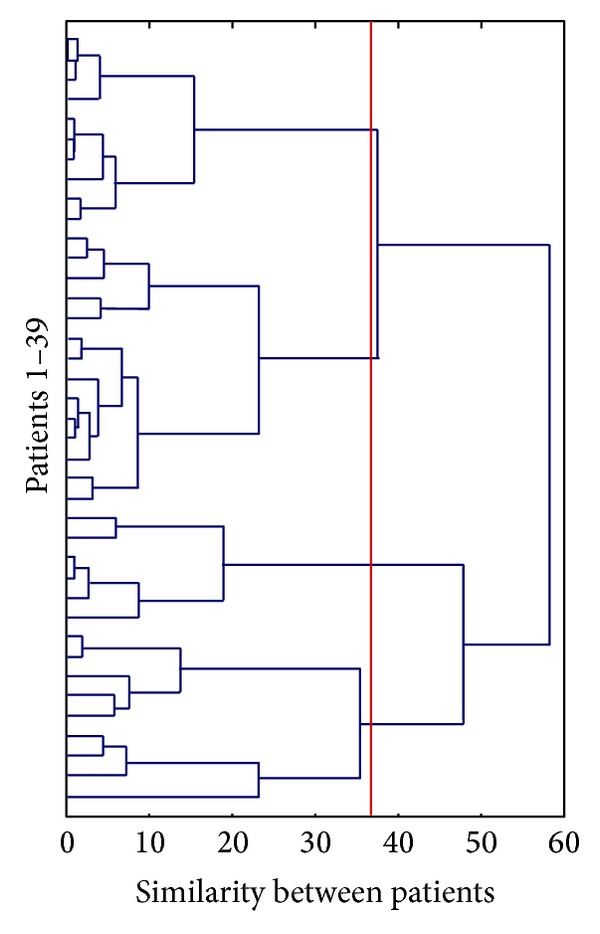
The dendrogram generated by the cluster analysis. Each patient (*N* = 39) begins as single clusters and step-by-step the most similar clusters are progressively joined together resulting at the end of the process (in the right-most part of the figure) in a single cluster grouping all the patients. The distance along the *x*-axis represents a measure of similarity between the patients; the vertical red line represents the linkage chosen to select the clusters.

In the hierarchical agglomerative cluster analysis individual patients begin as single clusters and step-by-step the most similar clusters are joined together resulting at the end of the process in a single cluster grouping all the patients. The choice of the number of cluster to extract is somewhat arbitrary. The agglomeration coefficients generated by cluster analysis revealed a demarcation point between four- and five-cluster solutions, suggesting that a four-cluster solution best distinguished the cases. A distinct grouping of all patients can be obtained by drawing a cut-point line along the dendrogram. An inspection of the clustering tree in Figure 1 confirms the four-cluster solution in which the cut-point chosen is represented as a vertical red line (Figure 1). These clusters are quite different as indicated by the horizontal distance one which needs to travel before the clusters are merged.

**Figure 2:**
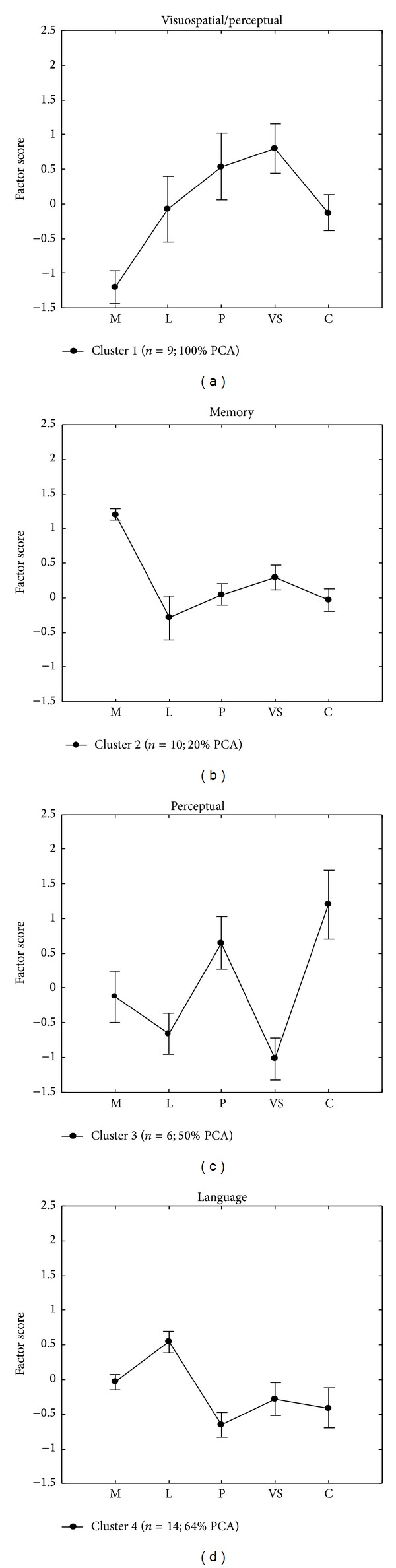
Plots (a)–(d) illustrate cluster 1–4. Mean factor scores and standard errors obtained by clusters, as a function of the factors extracted by the principal component analysis, are reported (M: memory, L: language, P: perceptual, VS: visuospatial/perceptual, C: calculation). High scores indicate lower performance. Each cluster number of patients and percentage of PCA are also reported.

The resultant four-cluster solution produced relatively well-sized groups labelled according to their most distinguishing characteristics (Figure 2). Higher scores indicate a larger deficit for the cognitive component measured by the factor relative to the other factors.

An analysis of variance across clusters with the 5 factors as repeated measures indicated a significant interaction cluster-by-factors (*F*_12,140_ = 9, *P* < .0001) with no significant main effects of cluster (*F*_3,35_ = 1.75, n.s.) and factors (*F*_12,140_ < 1). Fisher LSD post hoc comparisons were carried out to assess differences across clusters (Table 4) and between factors within each cluster (see text). Cluster 1 was composed by all clinically defined PCA patients (9/9), and cluster 2 had a clear majority of DAT patients (8/10), while cluster 3 (6 patients) and cluster 4 (14 patients) had a heterogeneous composition (Figure 2).

Cluster 1 was defined by good memory skills significantly different from weak performance in perceptual and visuospatial abilities and from language and calculation (all *P*s < .0005). Factor 3 and factor 4 (perceptual and visuospatial), as well as factor 2 and factor 5 (language and calculation), did not significantly differ from each other; however, the visuospatial abilities were significantly more impaired than language and calculation skills (*P*s < .05). Inspection of Figure 2 indicates that cluster 1 is specifically characterized by a visuospatial impairment relative to all the other clusters (cluster 1 will be referred to as visuospatial/perceptual). On the converse, cluster 2 was characterized by severe impairment of memory (all comparisons: *P*s < .005) while no other differences reached significance (all the other *P*s are n.s.) (hereafter memory). Cluster 3 was characterized by significant impairment in perception and calculation (hereafter perceptual) compared to all other factors under exam (all *P*s < .05), which in turn did not differ significantly from each other. Finally, cluster 4 was dominated by language disorders (hereafter language) (all *P*s < .005) associated with a memory deficit compared to perceptual abilities (*P*s < .05) and no other significant differences between the other factors.

Overall, a memory deficit is what mostly characterized cluster 2; instead a deficit in the visual (visuospatial ad visuoperceptual) domain, with memory preservation, characterized cluster 1. This was the only cluster in which a visuospatial deficit clearly emerged compared to the others; consistently, the majority of subjects with signs of left visuospatial neglect fell into this cluster. Cluster 3 had a perceptual impairment as cluster 1, but in this cluster the perceptual disorder was associated with a disorder in calculation. Finally, the involvement of language characterised cluster 4.

To evaluate the possible concurrent effect of other relevant variables in the emergence of the groups from the cluster analysis, we performed four separate one-way ANOVAs on the effect of age, education, disease duration (years), and general mental deterioration as assessed by MMSE; no significant difference emerged across clusters (*F*s were, respectively, 1.2; <1; 1.3; <1).

Cluster 1 (poor performance in “visual” domains) presented a prevalent right-sided parietal and temporal hypoperfusion; in cluster 2 (memory impairment) hypoperfusion was principally left temporoparietal and left frontal and in cluster 3 (perceptual and calculation disorders) mostly right posterior; cluster 4 (language deficit) presented a predominant temporal (>right), parietal (bilateral), and frontal (left) hypoperfusion.

## 4. Discussion

The cluster analysis generated four clusters of cognitive deficits, respectively, in the visuospatial/visuoperceptual, memory, perceptual/calculation, and language domain.

Cluster 1 and cluster 2 were consistent with two main phenotypes of AD pathology, DAT and PCA. The visuospatial deficit reached clear evidence in cluster 1, formed by a very homogeneous group of PCA patients, confirming that PCA is principally a dorsal stream pathology [3, 4, 7], although our results seem to confirm that perceptual deficits can cooccur [9].

Our data also suggest that AD-type pathology can generate subtypes with selective visuoperceptual and language deficits as well. The visuoperceptual deficit as a principal feature in PCA has been reported [10, 22] while the emergence of the pattern dominated by linguistic impairment was less expected in consideration of the selection criteria we adopted. This last finding would suggest that the language disorder is an important feature of AD pathology, and also when it does not emerge as a clinically evident aphasic syndrome.

The patterns of hypoperfusion were largely consistent with the cognitive impairments: confirming previous reports [23, 24] memory deficit was associated with hypoperfusion in the left temporoparietal and frontal regions; visuospatial and perceptual impairments were associated with a prevalent right parietal hypoperfusion. The right hemisphere plays a prominent role in controlling visuospatial attention [25]: six of our seven PCA patients with left side neglect were included in this cluster, confirming that a neglect is likely to emerge in neurodegenerative diseases for asymmetric involvement of the two hemispheres [26, 27]. The right posterior hypoperfusion in this cluster was also consistent with the perceptual impairment principally expressed by disorders in face processing [28]. When the deficits were limited to low level perception (X-detection) and calculation (cluster 3) we could confirm the involvement of the right posterior regions [10, 29]. Finally, when the language disorder was the principal feature (fluency, reading, and naming) hypoperfusion was bilateral temporoparietal and left frontal. While a bitemporal involvement is consistent with a possible disorder in object recognition [30] in confrontation naming tasks, the parietal damage could account for the reading deficit. Although dyslexia could be of perceptual nature, it is also possible that cluster 4 might have included patients with logopenic-type disorder in which the sublexical impairment is among the main features [31–33]. Thus, the patterns of hypoperfusion, even if collected using SPECT exams performed in the routine assessment, seem to well relate to the cognitive patterns extracted by the statistical analysis.

In conclusion, in our sample of patients, a purely data-driven analysis based on neuropsychological findings would confirm that PCA is principally a dorsal stream syndrome [3, 4, 7] distinct from typical amnestic AD. Nevertheless, our results suggest that AD-type pathology also generates other related, but distinct, syndromic subtypes, in which the visuoperceptual and language domains are specifically involved. These different patterns of cognitive deficits could reflect different vulnerability of specific functional networks to the same pathology.

## Supplementary Material

In supplementary material tasks used to explore a wide range of cognitive areas, and in particular visual perceptual and spatial domains, are reported.

## Figures and Tables

**Table 1:** Demographic and clinical data of DAT and PCA. Means (sd) and ranges are reported.

	Gender M/F	Age (y)	Education (y)	Duration illness (y)	Onset age (y)	CDR	MMSE
DAT	5/11	74.4 (5.8)	9.1 (4.5)	2.5 (0.6)	71.9 (5.8)	1.4 (1.0)	21.3 (3.7)
		61–86	5–18	1–3	59–83	0.5–3.0	17–26

PCA	5/18	72.4 (10.1)	8.1 (4.3)	2.3 (0.5)	69.7 (9.9)	1.1 (0.8)	20.8 (4.8)
		59–86	3–19	2-3	46–83	0.5–3	10–29

**Table 2:** Variables used for the principal component analysis. Means (sd) and ranges of the row scores obtained in DAT and PCA are reported.

Cognitive domains explored	DAT	PCA
Memory		
Rey immediate recall *N* = 75	17.7 (8.6) 6/34	24.7 (10.4) 9/44
Rey delayed recall *N* = 15	0.7 (1.6) 0/5	3.2 (2.8) 0/9
Rey recognition (0-1)	0.7 (0.1) 0.6/0.9	0.8 (0.1) 0.5/0.9
Babcock memory test (0–28)	2.1 (2.4) 7.3/0	3.7 (3.8) 0/11
Digit span forward (0–9)	4.9 (0.7) 4/6	5.1 (1.2) 4/9
Memory face *N* = 5	1.6 (1.2) 0/4	2.9 (1.4) 0/5
Visuoperceptual		
FEEST *N* = 60	37.1 (8.8) 20/51	32.1 (11.0) 0/53
Digital agnosia *N* = 5	3.2 (1.7) 0/5	2.0 (1.6) 0/5
Benton face recognition *N* = 52	34.4 (11.5) 19/44	25.3 (17.8) 0/47
Famous face recognition *N* = 32	24.4 (5.4) 14/30	18.3 (12.2) 0/32
Vosp X-detection *N* = 20	18.2 (1.9) 13/20	14.6 (5.6) 0/20
Color naming *N* = 5	4.2 (1.1) 1/5	4.3 (1.2) 0/5
Visuospatial		
Spatial span forward (0–9)	4.2 (1.0) 2/6	2.3 (2.0) 0/5
Double barrage (0-1)	0.9 (0.1) 0.5/1	0.7 (0.2) 0.5/1
Navon letters *N* = 30	18.4 (9.6) 7/30	16.3 (8.0) 7/30
Letter cancellation *N* = 104	99.9 (9.8) 64/104	72 (36.7) 0/104
Executive functions		
Rey's figure copy (0–36)	19 (12.7) 0/33	3.0 (5.1) 0/21
Language		
Letter fluency (F, A, S)	21.7 (13.9) 0/53	22.2 (13.4) 1/53
Semantic fluency	9.2 (4.3) 3/15	6.6 (3.7) 0/15
Reading *N* = 15	12.8 (3.3) 4/15	11.3 (3.8) 0/15
Writing *N* = 15	10.2 (2.7) 5/15	7.6 (4.0) 0/13
Object naming *N* = 28	15.5 (4.0) 6/20	13.1 (5.8) 0/20
Sentence comprehension *N* = 14	12.0 (2.5) 5/14	7.5 (5.0) 0/13
Calculation		
Addition *N* = 3	1.7 (1.1) 0/3	1.0 (1.0) 0/3
Subtraction *N* = 3	1.3 (1.1) 0/3	0.9 (1.0) 0/3
Multiplication *N* = 4	1.8 (1.4) 0/4	1.3 (1.4) 0/4

FEEST: facial expression of emotion: stimuli and tests; VOSP: visual object and space perception battery. For references see online resource.

**Table 3:** Factors extracted from the principal component analysis and variables that loaded into the factors.

(1) Memory	(2) Language	(3) Perceptual	(4) Visuospatial	(5) Calculation
Rey immediate recall	Semantic fluency	VOSP X-detection	Barrage	Additions
Rey delayed recall	Phonological fluency	Benton	Spatial span	Subtractions
Recognition accuracy	Reading	Famous faces	H cancellation	
	Naming			

**Table 4:** Post hoc comparisons (Fisher) across clusters (C) for each factor extracted.

Factors	C1	C2	C3	C4
Memory	C2***, C3*, C4**	C1***, C3**, C4***	C1*, C2**	C1***, C2***
Language	—	C4*	C4**	C2*, C3**
Perceptual	C4***	C4*	C4**	C1**, C2*, C3**
Visuospatial	C3***, C4**	C3**	C1***, C2**	C1**
Calculation	C3**	C3**	C1***, C2**, C4***	C3***

*P < .05; **P < .005, and ***P < .0005.
