# Supplementary material for: Posterior AD-Type Pathology: Cognitive Subtypes Emerging from a Cluster Analysis
Source: Behav Neurol. 2014 Jun 5;2014:259358. doi: 10.1155/2014/259358 (PMC4068066; doi:10.1155/2014/259358)
Supplement: Supplementary file 1 — In supplementary material tasks used to explore a wide range of cognitive areas, and in particular visual perceptual and spatial domains, are reported. [file 259358.f1.pdf]

SUPPLEMENTARY MATERIAL

The cognitive status was assessed by tasks exploring a wide range of cognitive domains: Mental Deterioration Battery (BDM) (revised version), Rey-Osterrieth complex figure, picture naming, Trail Making B, Babcock, aphasia and acalculia batteries (BADA, E.N.P.A.). The visual perceptual and spatial domains were explored by the Facial Expression of Emotion, unknown face identification, familiarity check and famous face recognition, Visual Object and Space Perception battery (VOSP), colour naming, Cortical Vision Screening Test (CORVIST), Navon's letter, letter cancellation, constructional apraxia, clock drawing, memory for faces and digital agnosia.

References

1. C. P. Hughes, L. Berg, W. L. Danziger, L. A. Coben, and R. L. Martin, "A new clinical scale for the staging of dementia," *British Journal of Psychiatry*, vol. 140, pp. 566-572, 1982.

2. M.F. Folstein, S. E. Folstein, and P.R. McHugh, "Mini-mental state: a practical method for grading the cognitive state of patients for the clinicians," *Journal of Psychiatric Research*, vol. 12, no.3, pp. 189-198, 1975.

3. G. A. Carlesimo, C. Caltagirone, and G. Gainotti, "The Mental Deterioration Battery: normative data, diagnostic reliability and qualitative analyses of cognitive impairment. The Group for the Standardization of the Mental Deterioration Battery," *European Neurology*, vol. 36, no. 6, pp. 378-384, 1996.

4. A. Rey, "L'examen psychologique dans les cas d'encephalopathie traumatique," *Archives de Psychologie*, vol. 28, pp. 215–285, 1941.

5. P. A. Osterrieth, “Filetest de copie d'une figure complex: Contribution a l'etude de la perception et de la mémoire, ” *Archives de Psychologie,* vol. 30, pp. 286–356, 1944.

6. M. Laiacona, R. Barbarotto, C. Trivelli, and E. Capitani, “Category-specific semantic defects: a standardised test with normative data,” *Archivio di Psicologia, Neurologia e Psichiatria,* vol. 54, no. 2, pp. 209-248, 1993.

7. G. Miceli, A. Laudanna, C. Burani, and R. Papasso, *Batteria per l’analisi dei deficit afasici (BADA)*, CEPSAG, Roma, Italy, 1994.

8. A. R. Giovagnoli, M. Del Pesce, S. Mascheroni, M. Simoncelli, M. Laiacona, and E. Capitani**,** “Trail making test: normative values from 287 normal adult controls,” *Italian Journal of Neurological Sciences*, vol. 17, no. 4, pp. 305-309, 1996.

9. H. Babcock and L. Levy*, Manual of directions for the revised examination of the measurement of efficiency of mental functioning,* Stoelting, Chicago, USA, 1940.

10. R. Capasso and G. Miceli, *Esame neuropsicologico per l’afasia (E.N.P.A.),* Springer Verlag, Italy, 2001.

11. A. Young, D. Perrett, A. Calder, R. Sprengelmeyer, and P. Ekman, *Facial Expression of Emotion: Stimuli and Tests (FEEST),* Thames Valley Test Company, Bury St Edmunds, UK, 2002.

12. A. L. Benton and M. E. Van Allen ME, “Impairment in facial recognition in patients with cerebral disease,” *Cortex,* vol. 4, no. 4, pp. 344-358, 1968.

13. P. Faglioni, A. N. Cremonini, and E. De Renzi, “Taratura su soggetti normali di facce sconosciute e familiari. Un contributo allo studio della prosopagnosia,” *Archivio di Psicologia Neurolologia e Psichiatria*, vol. 52, no. 3, pp. 339-350, 1991.

14. E. K. Warrington and M. James, *The Visual Object and Space Perception Battery (VOSP)*, Thames Valley Test Company, Bury St Edmunds, UK, 1991.

15. M. James, G. T. Plant , and E. K. Warrington, *Cortical Vision Screening Test*, Thames Valley Test Company, Bury St Edmunds, UK, 2001.

16. D. Navon, “Forest before trees: The precedence of global features in visual perception,” *Cognitive Psychology*, vol. 9, no. 3, pp. 353-383, 1977.

17. M. I. Freedman, I. Leach, E. Kaplan, G. Winocur, K. J. Shulman, and D. C. Delis, *Clock Drawing,* Oxford University Press, Oxford, UK, 1994.

18. B. A. Wilson, J. Cockburn, and A. Baddeley, *The Rivermead Behavioural Memory Test Manual*, Thames Valley Test Company, Bury St Edmunds, UK, 1985.

19. M. Brazzelli, E. Capitani, S. Della Sala, H. Spinnler, and M. Zuffi, “A neuropsychological instrument adding to the description of patients with suspected cortical dementia: the Milan overall

dementia assessment," *Journal of Neurology Neurosurgery and Psychiatry,* vol. 57, no. 12, pp. 1510-1517, 1994.
